# Transport of L-Arginine Related Cardiovascular Risk Markers

**DOI:** 10.3390/jcm9123975

**Published:** 2020-12-08

**Authors:** Sofna Banjarnahor, Roman N. Rodionov, Jörg König, Renke Maas

**Affiliations:** 1 Institute of Experimental and Clinical Pharmacology and Toxicology, Friedrich-Alexander-Universität Erlangen-Nürnberg, 91504 Erlangen, Germany; sofn001@lipi.go.id (S.B.); joerg.koenig@fau.de (J.K.); 2 Research Centre for Chemistry, Indonesian Institute of Sciences (LIPI), Kawasan PUSPIPTEK Serpong, 15314 Tangerang Selatan, Banten, Indonesia; 3 Division of Angiology, Department of Internal Medicine III, University Center for Vascular Medicine, Technische Universität Dresden, 01307 Dresden, Germany; Roman.Rodionov@uniklinikum-dresden.de; 4 College of Medicine and Public Health, Flinders University and Flinders Medical Centre, 5042 Adelaide, Australia

**Keywords:** transport, L-arginine, ADMA, SDMA, L-homoarginine

## Abstract

L-arginine and its derivatives, asymmetric and symmetric dimethylarginine (ADMA and SDMA) and L-homoarginine, have emerged as cardiovascular biomarkers linked to cardiovascular outcomes and various metabolic and functional pathways such as NO-mediated endothelial function. Cellular uptake and efflux of L-arginine and its derivatives are facilitated by transport proteins. In this respect the cationic amino acid transporters CAT1 and CAT2 (*SLC7A1* and *SLC7A2*) and the system y^+^L amino acid transporters (*SLC7A6* and *SLC7A7*) have been most extensively investigated, so far, but the number of transporters shown to mediate the transport of L-arginine and its derivatives is constantly increasing. In the present review we assess the growing body of evidence regarding the function, expression, and clinical relevance of these transporters and their possible relation to cardiovascular diseases.

## 1. Introduction

Cardiovascular diseases (CVD) remain the primary cause of mortality worldwide [1]. According to the World Health Organization (WHO), cardiovascular diseases are estimated to cause 17.9 million premature deaths worldwide annually. Inflammation, oxidative stress, and endothelial dysfunction which are strongly associated with various classical and non-classical CVD risk factors, are on the front line in the onset and progression of atherosclerosis and CVD in general.

Numerous independent risk markers for adverse cardiovascular outcomes have been identified so far. Nonetheless, only a few of them are considered established risk factors possibly suitable for therapeutic interventions. L-arginine-nitric oxide (NO) signaling plays a critical role in vascular function [2], a low plasma concentration of L-arginine results in impaired L-arginine-NO signaling and may promote endothelial dysfunction [3,4]. Therefore, L-arginine supplementation has been investigated as a possible therapeutic approach [5,6]. However, while preclinical and short-term interventions have shown promising results [7,8,9], the success remains somewhat limited in terms of long-term outcome and survival benefit so far [10]. Changes in the L-arginine plasma concentration have been associated with effects on nitric oxide synthase (NOS) activity that would not be expected based on the known affinity of endothelial NOS (eNOS/NOS3) for L-arginine. This has also been referred to as an “L-arginine paradox” [11], hinting at possible effects of inhibitors, such as asymmetric dimethylarginine (ADMA), or some form of compartmentalization [12,13,14].

Moreover, while L-arginine and its endogenous homologue L-homoarginine, which is also a substrate of nitric oxide synthase (NOS), have been investigated as a protective risk marker, the methylated L-arginine derivatives asymmetric and symmetric dimethylarginine (ADMA and symmetric dimethylarginine (SDMA)) have been studied as risk markers, which may impair L-arginine dependent pathways (for reviews see [15,16]). These markers show several interrelations of their biochemical pathways, but partly rather divergent (patho-)physiological associations with clinical outcomes.

In addition to the well-established role of L-arginine as a substrate for NO-mediated signaling [2], experimental and epidemiological data summarized in the following section indicate strong associations of its derivatives ADMA, SDMA, and L-homoarginine with cardiovascular function and cardiovascular outcomes. An understanding of the regulation of their plasma concentrations and homeostasis is essential to understand the interrelation and pathophysiological properties of these markers.

Vallance et al. demonstrated that accumulation of ADMA in renal failure or infusion of ADMA in healthy volunteers may impair NO-synthesis and endothelial function in renal failure [17]. Subsequently, ADMA was also found to be associated with hypertension [18,19]. Even though elevated plasma ADMA levels were reported in patients with chronic kidney diseases (CKD) [17], the role of impaired renal function in ADMA metabolism remains partly elusive [20,21]. Given the possible adverse effects of ADMA on endothelial (i.e., vascular) function and survival [22,23,24], an efficient ADMA clearance may represent a relevant mechanism to counteract its harmful effects.

ADMA is eliminated through intracellular enzymatic degradation mainly by dimethylarginine dimethylaminohydrolase 1 (DDAH1) [23,25], alanine-glyoxylate aminotransferase 2 (AGXT2) [26], and possibly some other mechanisms such as acetylation [27] and by renal excretion [28]; hence cellular uptake is essential for its degradation. Moreover, the important role of the liver in the elimination of ADMA may not only be attributable to the metabolism of ADMA, but also to transport protein-mediated excretion into the bile [29]. Despite its structural similarity to ADMA, SDMA appears not to directly interfere with NO-synthesis and endothelial function at physiological concentrations. Still, SDMA is independently associated with total mortality and adverse cardiovascular outcomes (discussed elsewhere in more detail [30]), leaving possible causal mechanisms to be elucidated. The independent association of L-homoarginine and renal function was found to be inverse to that of ADMA and SDMA [31,32,33,34,35]. L-homoarginine was also described to be a weak substrate of NOS and inhibitor of arginases. However, while biologically possible, these putative pathomechanisms may not completely explain the observations.

Moreover, as also detailed further below and evident from the data presented in Table 1, L-arginine and its derivatives ADMA, SDMA and L-homoarginine appear to be differentially handled in renal tubular cells, despite having relatively similar chemical properties, leading to marked increases and decreases, respectively, in their urinary concentration. Taken together, these findings suggest that distinct transport mechanisms for L-arginine and its related metabolites constitute a necessary prerequisite for compartmentalization and the differential handling and “uses” of these compounds by organs and tissues.

It is the focus of this review to provide insights into the transport of L-arginine and its derivatives. The following sections aim to provide an update on the transport protein-mediated cellular uptake and release of L-arginine and its derivatives, providing key characteristics of the major transport proteins involved, and, where available evidence linking these transport proteins to human disease with a special focus on CVD.

## 2. Transporter Families Shown to Transport L-Arginine and/or Its Derivatives

**Figure 1:**
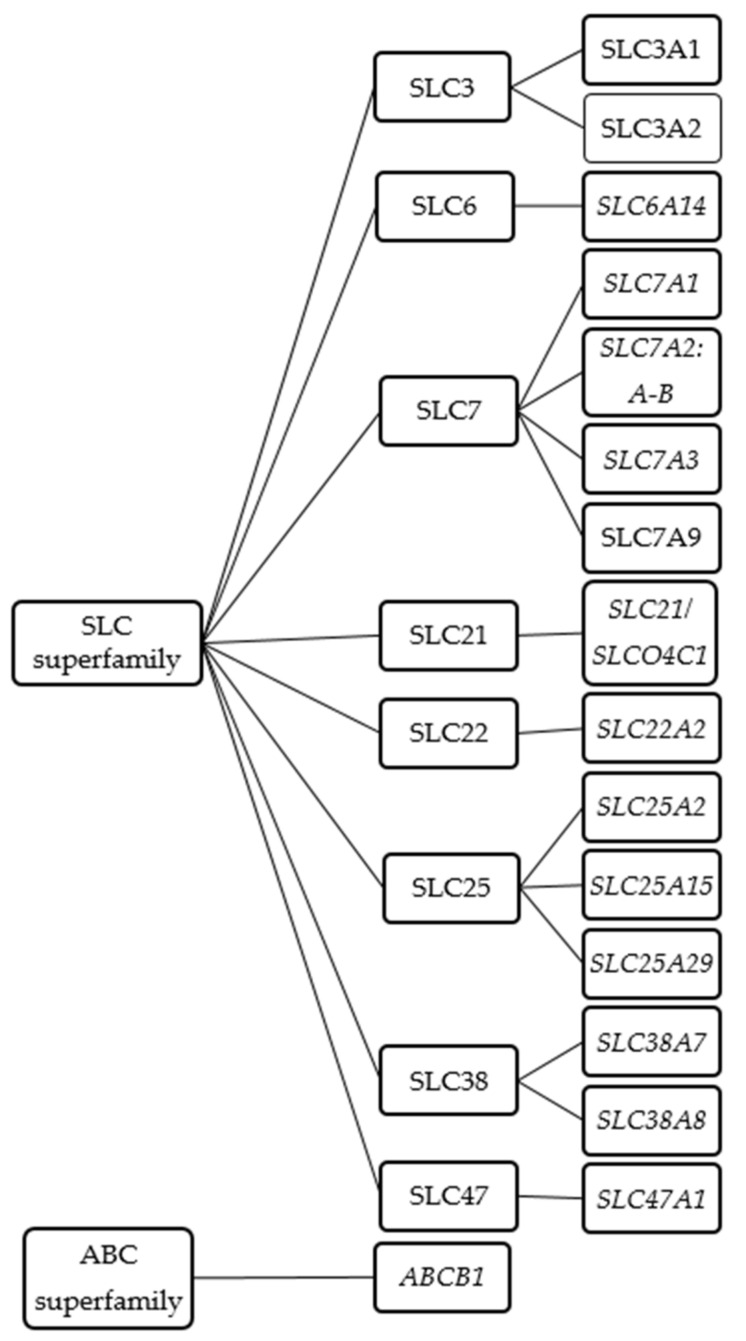
Schematic diagram of known L-arginine and its derivatives transporters.

As cationic amino acids, L-arginine and its derivatives do not readily diffuse across cell membranes but rather require some protein-mediated transport mechanism. In humans, at least 18 transporters are considered to mediate the exchange of L-arginine and its derivatives across the cell membrane or between the cytoplasm and cellular organelles such as the mitochondria or lysosomes. These transporters mostly belong to the solute carrier (SLC) superfamily. These include SLC3, SLC6, SLC7, SLCO/SLC21, SLC22, SLC25, SLC38, SLC47 (Figure 1 and Table 2). In addition, members of the adenosine triphosphate (ATP)-binding cassette (ABC) superfamily may also contribute to the cellular exchange of L-arginine and its derivatives (Figure 1 and Table 2). For practical reasons the order of presentation of the respective transporters will follow the transporter nomenclature rather than their biological function. 

### 2.1. The Solute Carrier (SLC) Superfamily

The SLC transporters (~400 genes) in mammalian cells consist of 65 distinct families, grouped based on their amino acid sequence (primary structure) and their transport function (a systematic record is provided by http://slc.bioparadigms.org/).

#### 2.1.1. SLC6 Family

The SLC6 family is the family of the sodium- and chloride-dependent neurotransmitter transporters. To date about 20 different *SLC6* genes have been identified in the human genome [36] that can be further classified into several branches (gamma amino butyric acid transporter branch, the monoamine transporter branch and the amino acid transporter branches 1 and 2) based upon their substrate specificity and sequence similarity [36]. The SLC6 family transport proteins accept a wide range of substrates including neurotransmitters, proteinogenic amino acids, betaine, taurine and creatine. Within this family, the neurotransmitter transporters were initially identified, and thus, it is recognized as the family of neurotransmitter sodium symporters (NSS) or the Na^+^/Cl^−^-dependent transporter family [37].

##### *SLC6A14* (ATB^0,+^)

ATB^0,+^ (Amino acid Transporter responsible for the activity of system B^0,+^), encoded by the gene *SLC6A14* (Table 2)*,* was functionally identified by Kekuda et al., and cloned from human choriocarcinoma and colon carcinoma [38]. Sloan et al. showed that the expression of *SLC6A14* mRNA was significantly higher in human lung and trachea than in other organs [39]. In addition, this sodium- and chloride-dependent neutral and basic amino acid transporter is expressed in pancreas, pituitary, skeletal muscle, placenta, human colon carcinoma cell lines [40,41], and intestine, colon, and kidney proximal tubules [39], where it is located in the luminal membrane [42].

This transporter recognizes neutral and cationic amino acids, excluding aspartate and glutamate [43]. It has some unique characteristics, which differ from other amino acid transporters. The transport activity is dependent on Na^+^/Cl^−^, highly concentrative, electrogenic [39], and influenced by the membrane potential [44,45]. So far, ATB^0,+^ is the only member of this family that has been identified to transport L-arginine. Studies in human bronchial epithelial cells reported K_M_ values of 80 μM for ATB^0,+^-mediated L-arginine uptake, which most likely is in the range of physiological relevance. The K_M_ value for L-arginine uptake by human ATB^0,+^ when expressed in *Xenopus oocytes* was reported to be 104 μM [39]. Issues with regard to the interpretation of K_M_ values obtained in different experimental settings will be addressed in the conclusion.

The *SLC6A14* gene has been linked to several diseases (e.g., obesity, ulcerative colitis, colon cancer, breast cancer, and cervical cancer) [46,47,48,49,50]. A meta-analysis of large genome-wide association studies (GWAS) identified the *SLC6A14* gene as a potential secondary modifier of cystic fibrosis (CF) comorbidities [51]. A recent study demonstrated that ATB^0,+^ plays a crucial role in L-arginine transport across the airway epithelial membrane [52]. In this ex vivo study, it was shown that inhibition of the ATB^0,+^ function in primary human respiratory cell cultures together with the *SLc6a14* gene knockout in mice resulted in increased L-arginine concentration in the airway surface liquid.

Interestingly, ATB^0,+^ expresses functional characteristics that may promote tumor growth: (1) Its ability to transport a large array of amino acids involved in various metabolic pathways vital for tumor growth. (2) Its capacity to facilitate amino acid uptake in a highly concentrative manner. Indeed, overexpression of *SLC6A14* has been reported in various cancers (e.g., colon cancer, breast cancer, pancreatic cancer, and cervical cancer) [47,48,50,53]. *SLc6a14*-knockout mice have been generated which do not express any visible physical or metabolic phenotype. However, development and growth of breast cancer were notably suppressed in a murine mammary tumor virus-polyoma middle tumor-antigen (MTV-PyMT) model deficient in *SLc6a14* as compared to MTV-PyMT mice expressing *SLc6a14* [54], indicating the potential tumor-promoting activity of ATB^0,+^ in the mammary gland. However, until now, no studies have been undertaken to investigate whether L-arginine derivatives (e.g., ADMA, SDMA, and L-homoarginine) are also recognized by this transporter. So far, there is no evidence for its involvement in NO-signaling or CVD.

#### 2.1.2. SLC7 Family

The SLC7 family (cationic amino acid transporter/glycoprotein-associated family) consists of two subgroups of transport proteins, cationic amino acid transporters (CATs) and heterodimeric (glycoprotein-associated) amino acid transporters (HATs) [55]. CATs are relatively selective for L-arginine and related compounds, including L-lysine and L-ornithine, whereas HATs have a broad(er) spectrum of substrates also including leucine, glutamine, tryptophan [56]. From the SLC7 family, CATs have been most extensively studied as a transport system for L-arginine and its derivatives. Five members of the CAT family have been cloned, so far. The transport is sodium- and pH-independent [57]. Several CATs have been shown to be sensitive to trans-stimulation [58]. CATs facilitate substrate accumulation against a concentration gradient because of a sensitivity to the membrane potential [59].

##### *SLC7A1* (CAT1)

Of the five CATs proteins, CAT1, a high-affinity electrogenic transporter, was the first to be described at a molecular level. It was initially cloned by Albritton et al. in search of the host cell protein accountable for infection by the murine ecotropic leukaemia virus (MuLV [60]). The *SLC7A1* gene encoding human CAT1, was mapped to chromosome 13q12-q14. Excluding the adult’s liver and lacrimal gland [61,62], relevant CAT1 expression can be observed in almost every cell or tissue, albeit at different expression levels (Table 3). CAT1 expression was shown to be modulated by several factors which include insulin, angiotensin II, hormones, nutrients, inflammatory cytokines, and amino acid starvation [63].

**Figure 2:**
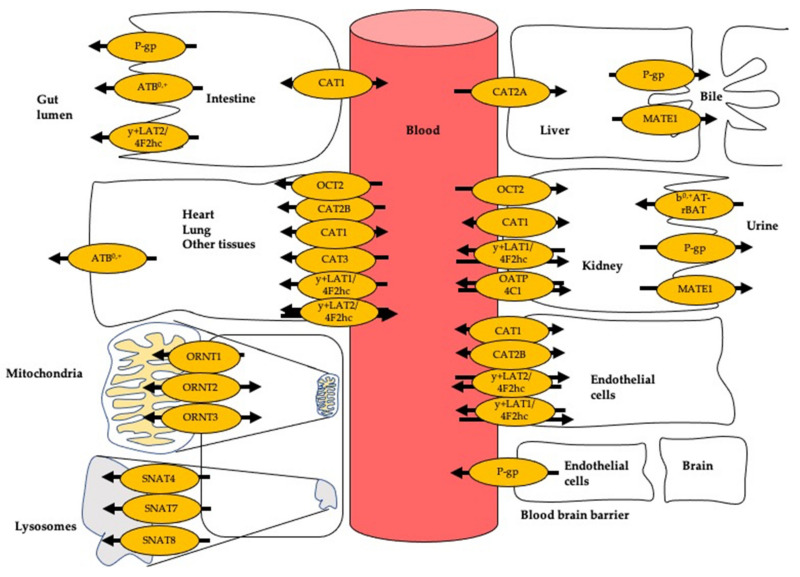
Transport of L-arginine and its derivatives in different cell types. Arrow indicates the direction of transport. ATB^0,+^: amino acid transporter responsible for the activity of system B^0,+^; CAT1: cationic amino acid transporter 1; CAT2A and CAT2B: cationic amino acid transporters 2A and 2B; MATE1: multidrug and toxin extrusion protein 1; OATP4C1: organic anion-transporting polypeptide 4C1; OCT2: organic cation transporter 2; ORNT1: ornithine transporter 1; ORNT2: ornithine transporter 2; ORNT3: ornithine transporter 3; P-gp: P-glycoprotein; SNAT7: sodium-coupled neutral amino acid transporter 4; SNAT7: sodium-coupled neutral amino acid transporter 7; SNAT8: sodium-coupled neutral amino acid transporter 8; y^+^LAT1: system y^+^L amino acid transporter 1; 4F2hc: 4F2 cell surface antigen heavy chain; y^+^LAT2: system y^+^L amino acid transporter 2.

By expression in *Xenopus oocytes,* it was shown that CAT1 facilitates the transport of cationic amino acids (CAAs) [64]. CAT1 transport activities depend on three different factors: concentration gradient, chemical gradient, and membrane potential [65]. CATs are sensitive to trans-stimulation, with CAT1 being the most pronounced [55,66,67]. This feature enables CATs to transport L-arginine and its derivatives both into and out of cells (Figure 2), leading to an exchange of cationic amino acids across the two sides of the membrane which depends on the presence of substrate on either side of the membrane (for review see [66]). Trans-stimulation increases the complexity of in vivo transport kinetics as the intra- and extracellular concentrations of the exchange partners and their respective transport mechanisms have to be considered as well as factors affecting the dynamic equilibrium of L-arginine and its derivatives across cellular membranes. The transport process is also electrogenic, involving the net movement of a positive charge. Based on data from in vitro studies it has been suggested that CAT1 is one major determinant of the plasma levels of L-arginine and its derivatives. CAT1 has been reported to be predominantly expressed in endothelial cells and is assumed to be responsible for 70–95% of L-arginine uptake [68].

CAT1 has a distinct feature that may enable this protein to play a significant role in endothelial function: it colocalizes with eNOS in endothelial cell caveolae. This colocalization may enable a direct local transfer of extracellular L-arginine by CAT1 to membrane-bound eNOS, thus making CAT1 a potentially selective supplier of L-arginine for eNOS [69]. CAT1 has mainly been studied as a L-arginine transporter, with early inhibition data indicating a likely role in the transport of ADMA and SDMA as well [70]. As a high-affinity transporter, CAT1 transports L-arginine with apparent K_M_ values in the range of 100 to 519 μM, depending on the model used [71,72]. In a human embryonic kidney (HEK) cell model the apparent K_M_ values for CAT1-mediated L-arginine influx was reported to be 519 μM and 183 and 175 μM for ADMA and L-homoarginine, respectively [71,73]. When interpreting the K_M_ values for the different substrates, their plasma or tissue concentrations have to be considered as well. Because in relation to typical plasma concentrations of 100 µM and 0.5 µM for L-arginine and ADMA, respectively, more L-arginine than ADMA may be transported by this transporter, in absolute as well as in relative terms, despite the transporter’s lower K_M_ for ADMA as compared to L-arginine. No direct studies have been reported for SDMA, so far, but inhibition of L-arginine transport by SDMA has competitive characteristics, indicating SDMA is a substrate as well [70,71]. The uptake of L-arginine was inhibited by ADMA and SDMA with half maximal inhibitory concentration (IC_50_) values of 758 and 789 μM, respectively [71]. L-homoarginine inhibited L-arginine transport with an IC_50_ of 1320 μM [73]. These IC_50_ values suggest that L-arginine transport is unlikely to be affected in a clinically important manner by physiological concentrations of ADMA, SDMA, and L-homoarginine.

The lack of CAT1 in homozygous knockout *SLc7a1* mice was found to be lethal [109]. Loss of function of L-arginine transport of up to 70% by small interfering RNA (siRNA)-mediated knockdown of *SLc7a1* resulted in delayed conceptus growth and abnormal function compared to the wild type [110]. These data suggest that at least in mice CAT1 is crucial during early development.

A study by Kakoki et al. linked rat CAT1 to hypertension [111]. They observed that infusion with an antisense oligonucleotide against CAT1 lead to a reduction of CAT1 expression in the renal medulla that was associated with decreased NO levels and development of hypertension in rats. In line with this Yang et al. observed an association of a single nucleotide polymorphism (SNP) ss52051869 (g.29514470G > A) in the 3′UTR of human *SLC7A1* with predisposition to essential hypertension in the Australian population [112]. Using *SLc7a1* transgenic mice the authors showed that overexpression *SLc7a1* results in significantly higher NO production and greater sensitivity of endothelial cells to acetylcholine compared with the wild-type mice. Later on, the same authors found that the altered *SLC7A1* expression was possibly mediated by disruption of miR-122 (micro RNA 122) binding [113]. Following this, a Finnish cohort study [114] reported another SNP in the *SLC7A1* gene (rs41318021; g.29514470G > A). Herein, it was reported that after a 15-year follow-up study, the CT or TT variants of the SNP were associated with a moderately higher predisposition to elevated diastolic blood pressure than the CC genotype.

However, in two independent clinical studies assessing whole blood transcriptome-wide gene expression, *SLC7A1* expression was found to be elevated in patients with atrial fibrillation [115,116]. In contrast to the SNP studies, associating SNPs related to impaired expression with hypertension, the elevation of expression in atrial fibrillation could also reflect a response to atrial fibrillation, rather than being its cause. Furthermore, several studies showed that *SLC7A1* expression can be modulated by amino acid supplementation, drugs or hormones, possibly mediating (adverse) drug effects.

Among others, cyclosporine A (CsA) was found to inhibit the cellular uptake of L-arginine by modulating CAT1 protein levels in human umbilical vein endothelial cells (HUVECs), which may result in impaired NO production and endothelial dysfunction [117]. The authors showed that CAT1 abundance was significantly reduced following a 48h incubation with CsA. These findings may explain some of the adverse cardiovascular effects of CsA, such as hypertension. Similarly, a study in HUVECs showed that progesterone inhibits arginine transport by modulating CAT1 expression via both protein kinase C-alpha (PKC α) and extracellular signal-regulated kinase 1/2 (ERK1/2) phosphorylation [118]. In contrast, CAT1 transport activity was found to be stimulated by estradiol through modulation of constitutive signaling transduction pathways involving extracellular signal-regulated kinase. These data may explain some of the observed effects of female sex hormones on the NO-mediated endothelial function [118].

Moreover, it has been reported that continued release of thyroid hormones may modulate mRNA expression of CAT1 through various mechanisms (e.g., integrin inhibition, genetic αv subunit down-regulation, or by phosphatidyl-inositol-3 kinase, mitogen-activated protein kinases, or intracellular calcium signaling inhibitors) which modulated L-arginine transport and results in altered endothelial production of NO and vascular tone. Thus, alterations of this mechanism may involve in some of the cardiovascular abnormalities in thyroid disorders [119]. Inflammatory mediators (e.g., tumor necrosis factor-α) (TNF-α) have been found to play a role in CAT1-mediated influx and efflux of L-arginine transport in endothelial cells [120].

Given the prominent role of CAT1 in cellular uptake and exchange of L-arginine and its emerging links to vascular function and hypertension (Table 4), CAT1 deserves further investigation not only as a target for adverse drug effects affecting CAT1-mediated uptake of L-arginine, but also as potential target to modulate the uptake and distribution of L-arginine and its derivatives in cardiovascular disease.

##### *SLC7A2* (CAT2A and CAT2B)

The human cationic amino acid transporter 2 (CAT2) was initially cloned from human intestine cDNA library [121]. The *SLC7A2* gene is located on chromosome 8p22. Alternative splicing of the *SLC7A2* gene leads to the expression of CAT2A and CAT2B comprising 657 and 658 amino acids, respectively. They vary in 19 residues in the region of transmembrane helices 8 and 9 [72]. Both transporters facilitate the cellular uptake and exchange of cationic amino acids, but differ in their tissue distribution and transport characteristics.

In contrast to CAT1, CAT2A and CAT2B have a more restricted expression pattern [61]. CAT2A is predominantly expressed in the liver, with additional, but much lower, expression in pancreas, cardiomyocytes, skeletal and vascular smooth muscle, and in cardiac endothelial cells [66]. From studies investigating cationic amino acid transport in mouse liver evidence emerged for the existence of a low-affinity but high-capacity transporter [122]. In line with this, HEK cells overexpressing human CAT2A show a matching low-affinity, high-capacity transport pattern (Table 3) [123].

The transport mechanisms of these CAT transporters have been studied in various experimental models. 

The affinity of human CAT2A for L-arginine and ADMA is relatively low, with K_M_ values of approximately 3000 μM and 4000 μM, respectively (Table 5) [72,123]. CAT2A transport is not saturated by L-homoarginine, whilst no direct data are available for the transport of SDMA by this transporter [73]. Of note, significant CAT2A-mediated uptake of both ADMA and L-arginine was detected only at high ADMA and L-arginine concentrations and not at physiological levels [123].

The cationic amino acid transporter 2 (CAT2B) is expressed as an inducible protein with a still low (K_M_ value of 952 μM) but slightly higher affinity for L-arginine than the one of CAT2A [123], despite its close similarity. A previous study from our laboratory using human embryonic kidney (HEK) cells overexpressing CAT2B demonstrated that CAT2B transported L-arginine and ADMA with K_M_ values of 952 and ≈4021 μM, respectively, and V_Max_ values of 15,300 and ≈14,300 nmol × mg protein^−1^ × min^−1^, respectively [123]. Similar to CAT2A, the CAT2B-mediated transport of SDMA has not been investigated yet.

The liver plays an important role, not only in L-arginine uptake and metabolism by arginases, but also in the absorption and metabolism of ADMA and SDMA from the circulation [29,127,128,129,130]. Altered, i.e., mostly elevated, plasma concentrations of L-arginine [131] and its methylated derivatives [132,133,134] have been reported in liver diseases, i.e., impaired liver function. So far, there is no direct evidence if high L-arginine or ADMA concentrations may affect CAT2A expression. Nevertheless, it can be assumed that in all of these cases, the ability of CAT2A to facilitate quantitative uptake of L-arginine and ADMA at high concentrations may play a role. As CAT2 mRNA is abundantly expressed in the liver, the low affinity but high capacity of CAT2A for L-arginine and related substrates [123] enables this organ to remove excess (post prandial) cationic amino acids from the blood without significantly competing with the uptake of theses substrates by transport systems of other tissues at lower concentrations [122]. 

While CAT1 is ubiquitously expressed [68], its extracellular L-arginine uptake is decreasing once intracellular cationic amino acids are depleted [67] (Table 4). Therefore, cells with higher arginine consumption (e.g., macrophages) [135] may induce CAT2B expression to provide immediate transport of L-arginine to meet functional needs. In line with this, a number of studies reported that CAT2B function is a potential target to modulate immunity [135,136,137,138]. Several mediators and drugs may stimulate the expression of CAT2B leading to increased cellular L-arginine uptake. Among others, rapamycin stimulates CAT2B mRNA and protein expression in HUVECs resulting in increased L-arginine transport [139]. Several inflammatory cytokines (e.g., interleukin 4 and interleukin 10, interferon gamma (IFN-γ) and tumor necrosis factor alpha (TNF-α) have been reported to stimulate expression and L-arginine transport in macrophages [140]. Moreover, TNF-α and supernatants of both *Pseudomonas aeruginosa* and *Staphylococcus aureus* also stimulate mRNA and protein expression of CAT2B and L-arginine influx in human corneal epithelial cells [141], suggesting that under inflammatory conditions, cells with inducible CAT2B may increase L-arginine availability particularly for iNOS.

In addition, CAT2 is also responsible for the transport of L-arginine in myeloid-derived suppressor cells (MDSC) and is an important modulator of the MDSC suppressive function of T cell response [the original publication does not distinguish CAT2A and CAT2B]. In mouse models of prostate-specific inflammation and cancer, CAT2 deficient-MDSC demonstrated significantly reduced capacity for modulating T cell responses due to diminished L-arginine supply for NO production [148]. In these models, it was shown that lack of CAT2 leads to enhanced antitumor activity. Furthermore, lack of CAT2 in murine models showed protective effects against hyperoxia-induced acute lung injury [149]. Considering the cardiopulmonary pathophysiological impacts of hyperoxia [150], lack of CAT2 may play a vital role in such conditions. 

In a recent cardiovascular health study, *SLC7A2* mRNA expression was found to be downregulated by the micro RNA hsa-miR-545 in myocardial infarction [142]. Moreover, in a GWAS study in Japanese population, a SNP (rs56335308; g.7561951: G > A) in the *SLC7A2* gene was found to be associated with plasma L-arginine concentrations [151]. However, it is still unclear whether these genetic variants directly modify the risk of CVD. In 2019, a first clinical case of mutations in the *SLC7A2* gene was reported [152]. Genetic analysis identified two loss-of-function mutations in the *SLC7A2* gene, which includes maternal allele deletion at c.874delA (p.Ile292Leufs*2) and one large genomic rearrangement encompassing exons 3 and 4 in the paternal allele. However, no data is available whether this loss of function is affecting either CAT2A or CAT2B or both proteins. The pathobiochemical phenotype was characterized by an increasing concentration of cationic amino acids in plasma and urine. However, it is yet unknown if this patient will develop cardiovascular diseases in a long-term follow-up, though the findings of this study suggest a possible reduction in iNOS activity. 

##### *SLC7A3* (CAT3)

The human cationic amino acid transporter 3 (CAT3), encoded by the *SLC7A3* gene, was initially cloned from human peripheral tissues [153]. This gene is located on X-chromosome (Xq13.1) encoding a 619-amino acid protein [153]. In rodents, CAT3 is considered as a brain-specific transporter. However, in humans the highest expression of mRNA has been reported in the thymus, followed by expression in the brain, uterus, mammary gland, testis, stomach, and ovary [153]. Moreover, CAT3 is highly expressed during fetal development, and predicted to be involved in embryogenesis and fetal development [154]. 

CAT3 can be kinetically distinguished from other members of the CAT family by a different affinity to L-lysine [154]. CAT3 mediates the sodium-independent transport of cationic amino acids with K_M_ values for L-arginine ranging from 40 to 120 µM (mouse CAT3) and 450 µM (human CAT3) [154,155]. It yet remains to be elucidated whether this transporter also transports ADMA, SDMA, and L-homoarginine. 

*SLC7A3* appears to be highly sensitive to genetic variations in humans, as documented by the low frequency of detrimental variants in available databases [156]. However, a rare genetic variant of the *SLC7A3* has been described in male individuals, and it has been suggested that in association with other genetic factors *SLC7A3* variants possibly contribute to the etiology of autism spectrum disorder (ASD) in male subjects [156]. These associations apart possible vascular effects have not been described yet. 

##### *SLC7A6* (y^+^LAT2) and *SLC7A7* (y^+^LAT1)

The *SLC7A6* (located on chromosome 16q22.1–16q22.2) and *SLC7A7* genes (located on chromosome 14q11.2) are members of the SLC7 family. *SLC7A6* (encoding for the y^+^LAT2 protein) and *SLC7A7* (encoding for the y^+^LAT1 protein) are similar in substrate selectivity and function. Both function as heterodimers, requiring the interaction between a “transporter” subunit and a chaperone subunit. The chaperone is responsible for the trafficking of the transporter to the plasma membrane [56]. The *SLC3A2* gene encoding the chaperone 4F2hc, consists of 529 amino acids and is also member of the SLC family and acts as chaperone for both of y^+^LAT2 and y^+^LAT1.

The y^+^L transporters are high-affinity transport systems for both cationic and neutral amino acids, with a unique feature of differential Na^+^-dependence: the influx of neutral amino acids is coupled to Na^+^ influx, whereas the efflux of cationic amino acids is independent to Na^+^ [157]. Both y^+^LAT1 and y^+^LAT2 are expressed in the intestine and in the kidney, where they localize to the basolateral membrane of absorptive epithelial cells (for a review see [158]). Several other tissues (e.g., brain, testis, parotid, heart, lung, and liver), were also reported to express these transporters [159,160,161,162]. Moreover, *SLC7A7* expression is also found in leucocytes, placenta, and lung [55].

These heterodimeric SLC transporters play a critical role in the release of absorbed cationic amino acids from the intestinal lumen and from kidney epithelial cells into the blood. They act as an exchanger by exporting cationic amino acids (e.g., L-arginine and its derivatives, L-ornithine, and L-lysine) into the blood in exchange for large neutral amino acids (e.g., glutamine, leucine) [55]. It has been shown that the system y^+^L facilitates L-arginine transport in human umbilical vein endothelial cells (HUVECs) with a K_M_ value of 42 μM [120]. However, these transport studies did not clearly distinguish between y^+^LAT1 and y^+^LAT2. Similar affinities were observed for L-arginine transport by human y^+^LAT1 (in monocyte-derived macrophages) and human y^+^LAT2 (in fibroblast) with K_M_ values of 182 and 145 μM, respectively (Table 2). To present, the transport of other L-arginine derivatives by both of y^+^LAT1 and y^+^LAT2 have not been studied.

**Figure 3:**
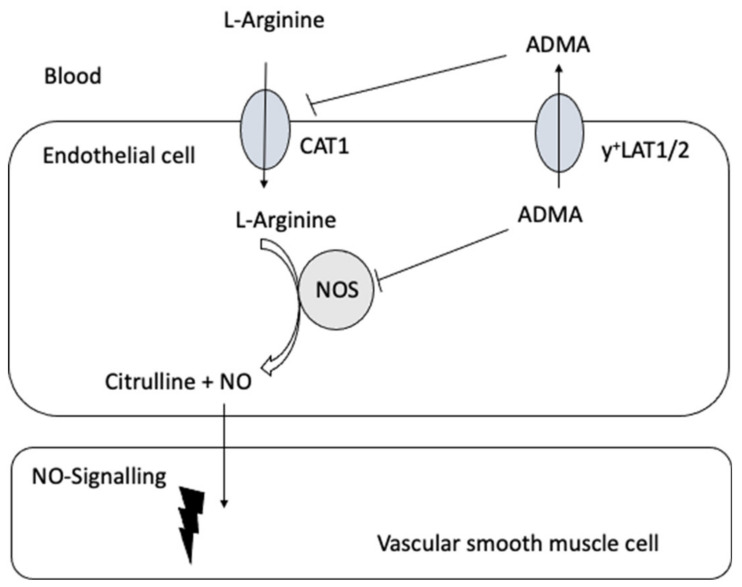
A schematic diagram of the possible interrelation of the transport of L-arginine and its derivatives and nitric oxide (NO) signaling in the vasculature.

Closs et al. [143] provided the first clinical evidence linking impaired expression of y^+^LAT1 to coronary and endothelial dysfunction resulting from an impaired cellular ADMA efflux and therefore intracellular ADMA accumulation (Figure 3 and Table 4). Oral L-arginine supplementation improved coronary and peripheral endothelial function and increased plasma ADMA concentrations. The latter was attributed to increased release of the elevated intracellular ADMA levels by alternative exchange transport systems (such as CATs) driven by the cationic amino acid L-arginine. These results suggest that under physiological conditions, y^+^LAT1 activity is fundamental in reducing excessive intracellular ADMA levels for the maintenance of endothelial function. Moreover, these data indicate that at least some forms of vascular disease involving endothelial dysfunction may be amendable to therapeutic interventions directed at defective cellular transport. 

It has been shown that inflammation is associated with an increased L-arginine transport mediated by y^+^LAT1. A number of proinflammatory cytokines, notably IFN-γ, have been described to exerts significant effects on the expression of y+LAT1 [163] but not y+LAT2, indicating that SLC7A7 is sensitive to IFN-γ. Furthermore, recent in vitro studies using astrocytes and rat brain endothelial cells demonstrated that y^+^LAT2 may have protective effects against hyperammonemic conditions in the brain [164]. In this study, ammonia-mediated up-regulation of y^+^LAT2 diminished NO synthesis by increasing intracellular ADMA and SDMA depletion without any significant changes in L-arginine concentrations. These in vitro findings suggest that y^+^LAT2 may play a role in the pathogenesis of hyperammonemic encephalopathies. 

Several studies indicate that genetic variations in the *SLC7A7* gene may result in a genetic predisposition for CVD [106,165]. In 2001, Kamada et al. demonstrated the effect of an L-arginine deficiency due to lysinuric protein intolerance (LPI) on vascular endothelial function in humans. In the reported case, the authors identified two mutations (5.3-kbp *Alu*–mediated deletion and IVS3+1 G→A) in the *SLC7A7* gene, which lead to a possible loss or a very low y^+^LAT1 activity [106]. Further analysis of these mutations revealed an insufficiency of brachial arterial dilatation after L-arginine infusion, low plasma L-arginine concentrations and impaired NO generation in endothelial cells. The authors also showed that following L-arginine supplementation, both NO generation and vascular endothelial function in the patient were normalized. Moreover, Kayanoki et al. report a marked endothelial dysfunction related to L-arginine deficiency in a patient later diagnosed with LPI [165]. In support of this, several studies reported that cardiovascular events are more prevalent in LPI patients [106,166,167], indicating that the *SLC7A7* gene is likely to be relevant in the pathogenesis of CVD.

##### *SLC7A9*-*SLC3A1* (b^0,+^AT-rBAT)

The heterodimeric b^0,+^AT-rBAT transporter encoded by the *SLC7A9* and *SLC3A1* genes, is also known as “cystine transporter”, based on its association with cystinuria detailed further below [168]. This protein was named based on its transport process; b^0,+^AT (“b” for broad; lowercase letter represent sodium independent active transport, and the superscript “0 and +” designating the net charge on the neutral and cationic amino acid substrates, respectively. Whereas, “r” in rBAT is referred to “**r**elated to BAT (BAT = **b**^0,+^ **a**mino acid **t**ransport)”. The primary transport activity is mediated by b^0,+^AT, while rBAT shows no transport activity and acts as a chaperone for directing b^0,+^AT to the correct plasma membrane. The rBAT (*SLc3a1* gene) was first isolated by expression cloning in *Xenopus oocytes* using both rat and rabbit kidney cDNAs [169,170,171]. The human *SLC3A1* gene was mapped to chromosome 2p21 [172]. The *SLC7A9* gene is located on chromosome 19, region q13.11 [173]. The b^0,+^AT-rBAT is a high affinity transporter for cationic amino acids and has a low affinity for neutral amino acids with different mode of Na^+^ dependency: the uptake of cationic amino acids is independent to Na^+^, whereas the efflux of neutral amino acids is Na^+-^dependent [174]. In humans, b^0,+^AT-rBAT proteins are predominantly expressed in the luminal membrane of kidney tubular cells of the kidney and enterocytes [175].

This protein complex facilitates the electrogenic transport of extracellular cystine and cationic amino acids for intracellular neutral amino acids [176]. On the intracellular site, the affinity for neutral amino acids is in the millimolar range [177]. In contrast, on the extracellular site, b^0,+^AT-rBAT has a high affinity for cationic amino acids and cystine (micromolar range) [177], which suggests a key role of this transporter in the renal reabsorption of cystine and cationic amino acids from the urine (Figure 2). It has been reported that the half-saturation constant for human L-arginine measured in different expression system was within the range of 100 μM to 180 μM [178,179,180] (Table 5). Whether b^0,+^AT-rBAT also transports ADMA, SDMA, and L-homoarginine remains to be elucidated. 

Inactivating mutations in the human *SLC7A9* and *SLC3A1* genes result in the partial failure of cationic amino acid reabsorption, clinically impressing as cystinuria. Cystinuric patients excrete increased amount of cystine and cationic amino acids with their urine [181]. Urinary excretion of L-arginine and L-homoarginine is increased by factors of one hundred-fold and ten-fold, respectively in patients with cystinuria while the plasma concentrations of L-arginine and L-homoarginine are significantly reduced compared to healthy subjects [100]. The heteromeric protein transporter b^0,+^AT-rBAT was found to be encoded by rather frequently mutated genes. 

So far, approximately 160 mutations have been identified in the *SLC3A1* gene and 116 mutations have been identified in the *SLC7A9* gene [182]. Most mutations identified in cystinuria are missense mutations leading to the change of a single amino acid in the protein. The effect of these mutations can range from having no effect on protein function to loss of function [183]. It is estimated that approximately 99% of cationic amino acids are reabsorbed at the apical membrane of proximal kidney tubules [96,97], indicating that b^0,+^AT-rBAT mediated transport may have a considerable leverage on the homeostasis of cationic amino acids. 

In a retrospective cohort study, which involved 442 patients with cystinuria an increased prevalence rate of hypertension with age was observed [144]. Another research group in a specialist cystinuria centre reported an overall prevalence of hypertension in subjects with cystinuria of 50.8% (61 out of 120 patients were hypertensive), with prevalence in males almost two-fold that of females (62.1% vs. 37.0%) [145]. When compared with the prevalence of hypertension in the normal healthy population, the prevalence of hypertension in cystinuric patients were 2-fold in men and 1.3-fold in women.

It was also shown that hypertension was associated with the stage of chronic kidney diseases (CKD); among the patients with normal renal function: 34.1% (10/29) were hypertensive, 50% (34/68) in the CKD stage 2, and 81% (17/21) of those with CKD stage 3 [145]. Taken together, these data support a role of *SLC7A9* and *SLC3A1* mutations in the genesis of hypertension. However, little is known regarding the precise underlying mechanisms. It would be of interest to evaluate plasma levels of L-arginine and its derivatives in relation to the long-term clinical outcome in patients with cystinuria.

#### 2.1.3. SLCO/SLC21 Family

The SLC21/SLCO family was initially named SLC21; however, the nomenclature of its members has been subjected to changes in 2004 based on phylogenetic relationships, and the family was designated to SLC21/SLCO, the solute carrier family of the organic anion transport polypeptides (OATPs) [239]. The SLC21/SLCO family represents a large group of proteins transporting a variety of substrates, including anionic, neutral and cationic compounds. Eleven different OATPs have been identified in humans of which, so far, (only) one member, the organic anion transport polypeptide 4C1 (OATP4C1), was found to be capable of transporting L-arginine and its derivatives [212]. 

##### *SLCO4C1* (OATP4C1)

The *SLCO4C1* gene encoding human OATP4C1 was mapped to chromosome 5q21.2 and encodes a protein with 724 amino acid residues. OATP4C1 is expressed in the basolateral membrane of the kidney proximal tubules [240]. This transporter facilitates Na^+^-independent uptake of a large variety of compounds and it is regarded as the first step in the vectorial transport (from blood across proximal tubule cells into urine) of these compounds (including uremic toxins) [241]. 

OATP4C1 mediates the transport of structurally diverse compounds including endogenous substances as such thyroid hormones (e.g., T3 and T4), cyclic AMP (cAMP) as well as drugs such as cardiac glycosides (e.g., digoxin and ouabain), methotrexate, and the dipeptidyl peptidase-4 inhibitor sitagliptin [240,242]. Although several members of the SLC21/SLCO family have been identified in detail, the functional characteristics of OATP4C1 still warrant further research. A recent study using stably-transfected HEK cells overexpressing human OATP4C1 showed that OATP4C1 can mediate the uptake as well as the efflux of L-arginine (uptake K_M_ of 48.1 μM) ADMA (uptake K_M_ value of 232.1 μM) and homoarginine (uptake K_M_ value of 49.9 µM) [212] (Table 5), suggesting that OATP4C1 may be involved in the excretion of uremic toxins in renal failure. Uptake of L-homoarginine could be inhibited by ADMA and SDMA with IC_50_ values of 117 and 54 μM, respectively. These inhibitory concentrations are well above the physiological range. A follow up study using double-transfected MDCK-OATP4C1-p-gp cells simultaneously expressing basolaterally localized OATP4C1 and luminally localized export pump P-glycoprotein demonstrated vectorial transport of L-arginine, L-homoarginine, and ADMA suggesting, that also this ABC-transporter may be important for their renal homeostasis of these compounds [185]. 

Altered OATP4C1 expression has been linked to CVD events [146,243] (Table 4) and endometrial cancer tissues [209], and genetic variants of the *SLCO4C1* gene were also found to be associated with obesity [244]. Increased expression of human OATP4C1 in a transgenic rat kidney model leads to an increased excretion of uremic toxins and reduced hypertension and cardiomegaly [146,243]. In addition, there is emerging evidence that genetic polymorphisms of *SLCO4C1* gene in patients with cardiac insufficiency contribute to renal clearance of digoxin. Based on a population pharmacokinetic model, *SLCO4C1* SNPs (rs3114660 and rs3114661) were associated with a significantly altered the renal clearance of digoxin [210]. 

#### 2.1.4. SLC22 Family

There are currently six subfamilies of SLC22 transporters, which have been predominantly studied as drug transporters, so far: (1) The organic cation transporter (OCT), (2) The organic cation/carnitine transporter (OCTN), (3) OCT/OCTN-related, (4) Organic anion transporter (OAT), (5) The OAT-like, and (6) The OAT-related [245]. Little is known about their role in the cellular uptake and release of endogenously formed molecules. As detailed below, recent studies suggest a contributing role of some of these transporters in the cellular handling of L-arginine and its derivatives [123]. 

##### *SLC22A2* (OCT2)

The organic cation transporter 2 (OCT2) is encoded by the *SLC22A2* gene located on chromosome 6q26. The protein is composed of 555 amino acid residues. This transporter is a low-affinity, high-capacity carrier that mediates the sodium-independent transport of cationic compounds [246]. OCT2 is strongly expressed in the proximal tubules of the kidney, located in the basolateral membrane in cells of the S2 and S3 region. Less abundant expression of OCT2 is also observed in the brain, the inner ear, the small intestine, and the lung [247,248,249,250]. 

Previously identified OCT2 substrates include tetraethylammonium (TEA), 1-methyl-4-phenylpyridinium (MPP^+^), the oral antidiabetic drug metformin, and cimetidine [251]. OCT2 facilitates the first step in the renal elimination of compounds and may control the intracellular concentrations of monoamine neurotransmitters in the brain [252]. It has been reported that the human OCT2-mediated L-arginine uptake may not be physiologically saturable (the predicted K_M_ value is higher than 10,000 μM). 

In addition, the Michaelis constant of ADMA for human OCT2 (K_M_: 900 ± 100 μM) [123] greatly exceeds physiological (about 0.4–0.9 μM) and even pathophysiological concentrations of ADMA (up to 8 μM in certain diseases) [253] in human plasma, indicating that human OCT2 could function as an ADMA transporter without saturation. This low-affinity transport of ADMA by OCT2 may contribute to the renal elimination of ADMA from circulation. No data regarding SDMA and L-homoarginine as substrates are available, so far.

Numerous polymorphisms in the *SLC22A2* gene have been identified [254]. However, most of these genetic variants have only been associated with drugs disposition and efficacy. In a recent meta-analysis using an integrated genome-transcriptome approach, *SLC22A2* gene expression was found to be significantly increased in pre-hypertensive groups [147] (Table 4). So far, the impact of *SLC22A2* SNPs on the renal clearance of uremic toxins such as ADMA has never been studied but, deserves to be further investigated.

#### 2.1.5. SLC25 Family

The SLC25 family is the largest SLC family in humans and composed of at least 53 family members. The SLC25 transporters mediate the transport of diverse substrates (e.g., various metabolites, nucleotides, and coenzymes) across the inner membrane of mitochondria. So far, approximately one-third of the putative mitochondrial transporters are “orphans”, with no known substrates [255]. However, until now, at least three transporters *SLC25A2*, *SLC25A15*, and *SLC25A29* are known to transport L-arginine and/or its derivatives [220,221].

##### *SLC25A2* (ORNT2), *SLC25A15* (ORNT1), and *SLC25A29* (ORNT3)

The *SLC25A2* and *SLC25A15* genes encode mitochondrial ornithine transporters 2 (ORNT2) and ornithine transporters 1 (ORNT1), respectively. ORNT1 was the first of the two isoforms to be identified and characterized [256], followed by the highly homologues ORNT2 [257]. Both proteins share 88% amino acid identity. The *SLC25A2* gene is located on chromosome 5q31.3 13q14.11 and the *SLC25A15* gene on chromosome 13q14.11 The product of the *SLC25A29* gene was firstly described as carnitine/acylcarnitine transporter-like (CACL) protein by Sekoguchi et al. [258]. Later, Camacho and Rioseco-Camacho classified this gene as ornithine transporter isoform 3 (ORNT3) because its overexpression retrieves the defect in ornithine uptake in cultured fibroblasts from patients with the hyperornithinemiahyperammonemia-homocitrullinuria (HHH) syndrome [223]. However, further study presented direct evidence that ORNT3 is a mitochondrial transporter responsible for the transport of basic amino acids in preference to L-arginine and L-lysine [227]. The *SLC25A29* gene, encoding ORNT3 is located on chromosome 14q32.2. 

ORNT2 was observed at the inner mitochondrial membrane in cells of several organs such as liver, testis, spleen, lung, pancreas, small intestine, and brain [259]. ORNT1 is expressed in mitochondria of liver, pancreas, lung, testis, small intestine, spleen, brain, and the heart [259]. Interestingly, *SLC25A2*mRNA is expressed in the human kidney whereas SLC25A15 could not be detected there [256]. ORNT3 is expressed in mitochondria located in the liver, in heart, in skeletal muscle, and in the brain/inner mitochondrial membrane [227]. 

These three mitochondrial transporters have at least several transport properties in common. They transport L-ornithine, L-lysine, L-arginine, and L-citrulline by homo or heteroexchange and unidirectionally, and they are inhibited by the same inhibitors [125]. Regarding L-arginine and its derivatives, human ORNT1 was reported to facilitate the transport of only L-arginine, so far (a K_M_ value for L-arginine is 1500 μM) [220]. In contrast to ORNT1, ORNT2 has a wider substrate specificity. ORNT2 also facilitates the transport D-isomers of ornithine, lysine, arginine, and citrulline and as well as histidine, L-homoarginine, and ADMA [260], which all can be metabolized in the mitochondria. The affinity of ORNT2 for L-arginine is approximately 710 μM [220,221]. ORNT2 facilitates both, the exchange of substrates as well as unidirectional transport. The reported initial rates of the L-arginine homoexchange and L-arginine/ADMA exchanges were 290 and 240 pmol × mg protein^−1^ × min^−1^, respectively; while the initial rates of the L-arginine as uniport was 50 pmol × mg protein^−1^ × min^−1^. Notably, the transport activities of the L-arginine/ADMA exchanges were similar to those of the arginine homoexchanges. The ORNT2-mediated ADMA transport was within the same range as the transport of the prototypic ORNT2 substrates known, so far, with a K_M_ value of 370 μM; and was highly specific, since symmetric dimethylarginine (SDMA), the structural isomer of ADMA, was not transported at all. Moreover, ADMA inhibited ORNT2-mediated transport of L-lysine with an inhibition constant of 380 μM, whereas SDMA, although not transported, showed some inhibition of ORNT2 transport activity [221].

Transport of ADMA could be a byproduct of mitochondrial L-arginine uptake. However, given that there are enzymes in the mitochondrial space which can metabolize ADMA, SDMA and L-homoarginine, it is likely that the transport of ADMA across the mitochondrial membrane could have different functions. The influx of ADMA into mitochondria could enable its degradation by mitochondrial alanine-glyoxylate aminotransferase 2 (AGXT2). Conversely, in cells lacking AGXT2 expression, ORNTs could mediate the mitochondrial efflux of ADMA produced by mitochondrial proteolysis for cytosolic degradation by DDAHs or for further efflux from the cell by transporters in the plasma membrane to allow degradation in other tissues [221]. L-homoarginine has also been reported to be transported by ORNT2, however, its transport kinetics remain to be defined [26,94,220]. 

The main function of the ornithine transporter 3 (ORNT3) is thought to facilitate the transport of L-arginine, L-lysine, L-histidine into mitochondria for mitochondrial protein synthesis. This transporter also recognizes L-homoarginine as a substrate [227]. The affinity of ORNT3 for L-arginine is moderate, with a K_M_ value of 420 μM [220,225]. Porcelli et al. demonstrated that ORNT3-reconstituted liposomes take up external L-arginine more efficiently in the presence of internal L-arginine, L-lysine, and L-homoarginine compared to ornithine, histidine, or carnitine, or compared to the absence of any internal substrate [227]. This transporter mediates the transport of L-arginine in two different ways, as exchange (homo or heteroexchange) or as uniport. The transport affinity for L-arginine as exchange (homo/heteroexchange) or as uniport is rather similar, with K_M_ values of 420 μM and 470 μM, respectively. By contrast, the average V_Max_ of L-arginine uptake measured as homo/heteroexchange is 3.1-fold higher than that derived from uniport, i.e., 237 pmol × mg protein^−1^ × min^−1^. This transporter therefore can mediate either L-arginine uptake or release. It was shown that L-homoarginine can competitively inhibit L-arginine exchange with an inhibitory constant (K_i_) value of 450 μM, however no clear data is available regarding the transport kinetics of L-homoarginine [227]. To present, no data have been reported related to the ORNT3-mediated transport of ADMA and SDMA.

Until now, no genetic variations in the human *SLC25A29* gene have been identified that link it to any diseases. In contrast, genetic variations in the *SLC25A2* and *SLC25A15* genes have been shown to be responsible for the hyperornithinemia-hyperammonemia-homocitrullinuria (HHH) syndrome [256]. However, this rare syndrome is beyond the scope of this review. In the context of CVD, no studies have been done related to the role of mitochondrial transporters, in this respect it is of interest, that *SLC25A2* likely plays an important role in regulating ADMA at the mitochondrial level. As recent studies highlight the role of mitochondrial AGXT2 in the metabolism of ADMA and L-homoarginine [26,94], the mitochondrial transport of these substrates certainly warrants further investigation.

#### 2.1.6. SLC38 Family

The SLC38 (System A and System N sodium-coupled neutral amino acid transporter) family is composed of 11 members, encoded by the genes *SLC38A1*-*11* [125]. The members of this group are characterized as **s**odium-coupled **n**eutral **a**mino acid **t**ransporters (SNATs) [261] with glutamine as preferred substrate throughout the family. So far three human SNATs are known to transport L-arginine [229,230,232]: SLC38A4 (SNAT4), SLC38A7 (SNAT7), and SLC38A8 (SNAT8).

##### *SLC38A4* (SNAT4), *SLC38A7* (SNAT7), and *SLC38A8* (SNAT8)

The *SLC38A4* gene, encoding for the SNAT4 protein, is located on chromosome 12q13 and the protein consists of 547 amino acids. This transporter is related to the members of the glutamine transporter family. SNAT4 is expressed in the liver [229] and in the placenta [262]. The *SLC38A7* gene (encoding SNAT7 protein) and *SLC38A8* gene (encoding SNAT8 protein) are clustered together in the chromosomal region 16q21 and q23.3, respectively. Both, SNAT7 and SNAT8, are expressed predominantly in the central nervous system, almost exclusively in neurons [230,232]. Both of these transporters mediate Na^+^-dependent influx and efflux of small neutral amino acids [125].

In humans, the sodium-coupled neutral amino acid transporter 4 (SNAT4) facilitates the transport of neutral amino acids and K-(methylamino) isobutyric acid (MeAIB) in a Na^+^-dependent manner, and the transport of cationic amino acids in a Na^+^-independent manner [125]. This transporter has a higher affinity for cationic amino acids (K_M_: 300 μM for L-arginine) compared to neutral amino acids (K_M_: 1600 μM for L-glycine), which is similar to that of y^+^LATs [229]. The transport of other L-arginine derivatives (ADMA, SDMA, and L-homoarginine) by this transporter has not been reported yet.

SNAT7 and SNAT8 are sodium coupled (system N) transporters of the SLC38 family and known as lysosomal glutamine transporters. In a *Xenopus oocyte* model overexpression of SNAT7 was associated with a significant increase of L-arginine uptake [230]. Similarly, SNAT8 also showed a high preference for L-glutamine, L-alanine, L-histidine, L-aspartate and L-arginine [232]. Similar to SNAT4, the transport of ADMA, SDMA, and L-homoarginine by these two transporters remain to be investigated.

While CAT2A is a low-affinity transporter with a K_M_ value for L-arginine within the millimolar range, SNAT4 has an about 10-times higher affinity for L-arginine (K_M_: 300 μM) [229]. Since SNAT4 is expressed abundantly in the liver, it is assumed that this transporter largely contributes to the cellular uptake of L-arginine into hepatocytes under physiological conditions [229]. However, if SNAT4 contributes relevantly to the total uptake of L-arginine into the liver remains doubtful for at least one reason: the liver expresses highly levels of arginase 1 which catalyzes the hydrolysis of L-arginine to urea and L-ornithine [263]. Thus, the functionally significant presence of a high-affinity L-arginine transporter (in addition to the abundant expression of CAT2) would increase the amount of L-arginine taken up by the liver and limit the availability of arginine for other pathways including NOS signaling. 

Among the three transporters mentioned here, the consequences of gene deletion are known only for murine *SLc38a4* [226]. The paternal knockout of *SLc38a4* shows an intrauterine growth retardation. In humans a SNP of *SLC38A8* (c.1234G>A[p.Gly412Arg]) was found to be associated with foveal hypoplasia [231]. It remains to be elucidated whether these three members of SLC38 family also have a function in the cardiovascular system related to L-arginine and its derivatives. 

#### 2.1.7. The SLC47 Family

The SLC47 family is composed of two human members MATE1 (SLC47A1) and MATE2-K (SLC47A2). At present, L-arginine and ADMA transport has been characterized only for MATE1 126]. 

##### *SLC47A1* (MATE1)

The multidrug and toxin extrusion protein 1 (MATE1) is a 570 amino acid integral membrane protein encoded by the *SLC47A1* gene located on chromosome 17p11.2. In humans, transcripts of MATE1 are ubiquitously expressed in the body, including kidney, liver, brain, colon, lung, placenta, and skeletal muscle [264]. However, at protein level, MATE1 could be detected in kidney and liver (located in the luminal membrane of proximal tubular cells and the canalicular membranes of hepatocytes) [265,266]. Functionally, MATE1 is an electroneutral, sodium-independent, and pH-dependent proton antiporter for organic compounds [267]. 

MATE1 mediates the bidirectional transport of organic cations and depends on a proton gradient. Thus, this protein may function as an uptake or efflux transporter depending on the orientation of the proton gradient [268,269]. MATE1 recognizes various organic cations, some non-charged compounds, and some zwitterions as substrates [266]. Various endogenous compounds, such as creatinine, thiamine, guanidine, and estrone-3-sulfate, were identified as human MATE1 substrates [266]. 

A recent study showed that MATE1 can mediate the uptake of L-arginine and ADMA [123]. The uptake of L-arginine and ADMA is pH-dependent, with uptake of L-arginine and ADMA markedly increased following alkalinization of the extracellular medium [123]. In this study, MATE1 facilitated the cellular uptake of L-arginine and ADMA with a rather low activity and uptake ratios at pH 7.3 of only 1.2 and 1.1, respectively (Table 5). It is still of interest that ADMA can be recycled to L-citrulline in the kidney [270]. To date, the MATE1-mediated transport properties of SDMA and L-homoarginine are unknown. 

The uptake (with subsequent metabolism) or excretion of ADMA by MATE1 may be rather unspecific, attributable to its broad substrate spectrum, it may, however, under certain circumstances contribute to the renal elimination of ADMA. With respect to CVD its role in the renal excretion of xenobiotics, including drugs may be of more relevance, though. 

### 2.2. The Adenosine Triphosphate (ATP)-Binding Cassette (ABC) Superfamily

The ATP-binding cassette (ABC) superfamily consists of two types of proteins, the cytosolic ABC-ATPases and the ABC transporters. The major part of the ABC transporters is located at the plasma membrane and actively transports a wide array of substrates with hydrolysis of adenosine triphosphate (ATP) as the driving force. There are currently at least 50 known human ABC transporters classified into seven groups from ABCA to ABCG. The vast majority of these transporters are expressed as exporters facilitating the efflux of a variety of substrates. The ABCB subfamily consists of 11 members. Its most prominent member is likely *ABCB1* it is also known as multidrug resistance protein or permeability-glycoprotein (P-glycoprotein, P-gp). To date, only *ABCB1* has been shown to mediate the transport of L-arginine and its derivatives [213]. 

#### ABCB1/MDR1 (P-glycoprotein)

P-glycoprotein (P-gp) is an active membrane bound efflux transport protein pump. It was first identified in the membranes of cancer cells, where it contributed to the phenomenon of multidrug resistance [271]. The *ABCB1* gene is located on chromosome 7q21.12 and encodes 1280 amino acid residues. Besides being prominently expressed in the apical membrane of epithelial cells in the intestine and in the kidney proximal tubules [272], *ABCB1* is also expressed in the biliary membrane of hepatocytes and numerous other tissues (apical surface of brain capillary endothelial cells, adrenal gland, pancreatic ductile, and placental trophoblast, and in the arterioles and capillaries of the left ventricular myocardium) [273]. A signature role of this adenosine triphosphate (ATP)-driven transport protein is its ability to recognize a variety of structurally diverse molecules ranging in size from 100 to 4000 daltons (Da) (molecular mass) [274].

P-gp is mostly known as an efflux pump protein for a wide range of drugs: [275,276]. The function of P-glycoprotein (P-gp) can be categorized according to its anatomical localization (Figure 2): P-gp limits xenobiotic (i.e., drug) absorption in intestinal tissues; P-gp protects sensitive organs and tissues (i.e., the brain) by extruding its substrates from the cells or tissues into the circulation. Furthermore, P-gp also facilitates clearance of drugs and metabolites into bile and urine [277]. In the kidney, P-gp is specifically expressed at the side of the tubular apical membrane, limiting the systemic exposure to xenobiotics by extruding the molecules from the epithelial cells to the luminal space [278].

P-gp contribution to the transport of L-arginine and SDMA has never been studied before, yet, it was only recently that ADMA and L-homoarginine had been associated with P-gp [213]. This ATP-driven transporter paired with the *SLCO4C1* transporter was reported to facilitate the efflux of ADMA and L-homoarginine through a transcellular transport (from the basolateral to the apical compartment) with transport ratios of 2.0- and 3.4-fold, respectively.

As with other transport proteins with broad spectrum of substrates, transport of L-homoarginine could simply be attributed to unspecific activity. However, given its broad expression it is likely that P-gp contributes to the transport and regulation of both L-arginine derivatives. Apart from its role in the plasma disposition of aldosterone [279,280,281], no obvious associations between P-gp activity towards biomarkers and cardiovascular disease have been reported yet.

## 3. Conclusions

L-arginine is the key substrate for NO-mediated signaling in the cardiovascular system and the plasma concentrations of its derivatives L-homoarginine, ADMA and SDMA are independently associated with cardiovascular outcomes and mortality. However, despite the substantial evidence linking these substances more or less directly to cardiovascular physiology and outcomes their functional interrelation remains only partially understood, leaving the question unsettled where the “risk marker” ends and rather the “risk factor” begins. Here a better understating of the transport mechanisms governing the cellular uptake and exchange of these substances may provide some necessary insights, which are currently lacking.

From the evidence discussed, the identification of distinct transport proteins that facilitate the transport of L-arginine and its derivatives will likely improve our understanding on the effects of L-arginine and its derivatives in maintaining vascular health. The identification of the truly relevant transport systems, with respect to cardiovascular disease is far from easy, though. Especially as the number of transporter proteins found to transport L-arginine and its derivatives is continuously growing, with about 18 human transporters identified so far.

In principle, lower K_M_ values indicate higher affinities and it is tempting to directly compare K_M_ values reported in different publications. However, before doing so, methodological issues should be carefully considered. Difficulties in determining the contributions of an individual transport protein often arise from the redundancy of transport systems for amino acids and related compounds in most tissues, with multiple alternative transport proteins for a single substrate available. For this reason, often only “apparent” rather than absolute transport activities can be provided for living cells, because it is practically impossible to block endogenous transport activity except for that of interest. From a biological perspective, these redundancies may also have advantages, as they would render the system less sensitive to impairment of an individual transport protein. The term “apparent” also indicates the K_M_ and V_Max_ values should be taken with a grain of salt, especially when comparing K_M_ values of different transporters which are often obtained in different experimental settings.

Given the importance of substrates like L-arginine for cellular function, these redundancies are not surprising. However, redundant (i.e., “background “) transport by different transport systems puts some limits the characterization of the contribution of individual transport proteins in all but the most artificial experimental settings, which in turn come with other limitations. Furthermore, molecules with divergent pathophysiological properties such as L-arginine and ADMA share major transport systems, making it difficult to gauge the net in vivo impact of an individual transport system in CVD. 

Despite all these limitations, the largely “apparent” transport data obtained from cell lines overexpressing transporters of interest already allow substantial insights regarding the putative functional role of the transporters with regard to the respective substrates and possible impact on cardiovascular physiology. Based on the data available so far, members of the CAT and LAT family such as CAT1 and y^+^LAT1/2 can be considered as major players, especially with respect to endothelial function and cardiovascular disease. Furthermore, case report data indicate that a y^+^LAT1 related transport deficiency may be corrected by L-arginine supplementation treatment [143]. 

Studies of human diseases, animal models, and cultured cells have demonstrated that there are at least two different functions through which L-arginine and its derivatives transporters can be related to CVD. Firstly, by providing adequate L-arginine for NO production. Secondly, by contributing to the disposition and homeostasis of L-arginine and its derivatives. It will be a key issue for future research in this area to identify the transport proteins and/or molecular mechanisms mediating differential transport of the chemically very similar substrates (L-arginine and L-homoarginine as compared to ADMA and SDMA). The relative changes in the concentration ratio of L-arginine and SDMA between plasma and urine are highly suggestive of a transport system that allows the selective depletion of L-arginine and L-homoarginine from the urine while SDMA is excreted into the urine. These chemically similar substances may actually turn out to be useful tools for the dissection of differential transport mechanisms. However, given the much higher plasma and tissue concentration of L-arginine, any apparently differential transport of L-homoarginine or ADMA and SDMA may simply reflect differences in substrate concentration than evolved differences in substrate specificity of the respective transporter.

Finally, drugs may affect the transport of L-arginine and its derivatives, possibly contributing to adverse as well as beneficial effects in CVD. Alternatively, selective inhibition of (re-)absorption of ADMA and SDMA at sites like the small intestine or the kidney could enhance their elimination. Conversely, selective inhibition of L-arginine and L-homoarginine uptake by tissues primarily involved in their degradation and elimination could increase their availability for tissues where they support protective functions, such as endothelial function. It remains to be shown, however, that the latter two mechanisms could also work in humans.

## Figures and Tables

**Table 1:** Physiological characteristics of L-arginine and its derivatives.

	L-Arginine	Asymmetric Dimethylarginine (ADMA)	Symmetric Dimethylarginine (SDMA)	L-Homoarginine
Plasma concentration Reference values in healthy populations (2.5th–97.5th centile)	41–114 µM [74]; 72.4–113.7 µM [75,76]	0.41–0.96 µM [77]	0.27–0.67 µM [77]	1.6–2.6 µM [77]; 1.4–2.5 µM [78,79,80,81]
Source/synthesis	Diet (85–90%; ca. 5 g/day) [82] and 10–15% endogenous synthesis in the kidney (16 μM/kg/hr) [83,84]	Endogenous hydrolysis of proteins containing asymmetrically methylated L-arginine residues [85]; possible contribution of diet [86]	Endogenous hydrolysis of proteins containing symmetrically methylated L-arginine residues [85]; possible contribution of diet [86]	Endogenous synthesis by glycine amidino transferase (AGAT) [87]; possible contribution of diet [88]
Metabolism	Major enzymes: L-arginine: glycine amidino transferase (AGAT), NO synthases (NOS; 3 isozymes), arginases (2 isozymes), and L-arginine decarboxylase [89]	Dimethylarginine dimethylaminohydrolase 1 (DDAH1) accounts for > 80% of the metabolic elimination [25]; Dimethylarginine dimethylaminohydrolase 1 (DDAH2) [90]; AGXT2 [26]	Primarily by renal excretion [91]; AGXT2 [92,93]	Metabolized by alanine—glyoxylate aminotransferase 2 (AGXT2) [94]; arginase and NO-Synthases [95] (relative contribution are uncertain)
Renal clearance	~0.14 mL/min [96,97,98,99,100]	~69 mL/min [101]	~71 mL/min [101]	~0.7 mL/min [78,79,80,81,102]
Association of plasma concentration and renal clearance	Positive correlation with estimated glomerular filtration rate (eGFR) [74]	Weak inverse correlation with eGFR [103]	Strong inverse correlation with eGFR, like creatinine [103]	Positive correlation with eGFR [104]
Association with mortality and cardiovascular disease	In most studies no independent biomarker for cardiovascular events or total mortality [105]; In short term supplementation studies associated with functional improvements of endothelial function [106]	Elevated plasma concentration independently predicts total and cardiovascular mortality [30]	Elevated plasma concentration independently predicts total and cardiovascular mortality [30]	Low plasma concentration independently predicts total and cardiovascular mortality [33,107,108]

**Table 2:** Nomenclature of transporters of L-arginine and its derivatives.

Gene Human/Rodent.	Protein	Alternative Protein Name, (Assoc. with)	Transport Type
*SLC3A1/SLc3a1*	rBAT	NBAT	Chaperone (not transporting)
*SLC3A2/SLc3a2*	4F2hc	CD98hc, FRP	Chaperone (not transporting)
*SLC6A14/SLc6a14*	ATB^0,+^	β-alanine carrier	F
*SLC7A1/SLc7a1*	CAT1	System y^+^, ATRC1	F (non-obligatory E)
*SLC7A2/Slc7a2*	CAT2A & CAT2B	System y^+^, ATRC2	F (non-obligatory E)
*SLC7A3/SLc7a3*	CAT3	System y^+^, ATRC3	F (non-obligatory E)
*SLC7A6/SLc7a6*	y^+^LAT2	System y^+^L [4F2hc]	E (intracellular cationic amino acids/Na^+^-independent against extracellular large neutral amino acids/Na^+^-dependent)
*SLC7A7*	y^+^LAT1	System y^+^L [4F2hc]	E (intracellular cationic amino acids/Na^+^-independent against extracellular large neutral amino acids/Na^+^-dependent)
*SLC7A9/SLc7a9*	b^0,+^AT	System b^0,+^ [rBAT]	E (preferentially extracellular cationic amino acid and cystine against intracellular neutral amino acid)
*SLCO4C1/Slco4c1*	OATP4C1	SLC21A2, OATP4C1, OATPX, OATP-H	F
*SLC22A2/SLc22a2*	OCT2	None	F
*SLC25A2/SLc25a2*	ORNT2	ORCT2	F (non-obligatory E: homoexchange, heteroexchange, unidirectional)
*SLC25A15/SLc25a15*	ORNT1	ORC1	F (non-obligatory E: homoexchange, heteroexchange, unidirectional)
*SLC25A29*	ORNT3	ORC3	F (non-obligatory E: homoexchange, heteroexchange, unidirectional)
*SLC38A4/SLc38a4*	SNAT4	ATA3	F
*SLC38A7*	SNAT7	None	F
*SLC38A8*	SNAT8	None	F
*SLC47A1*	MATE1	None	E
*ABCB1*	P-gp	None	F

Detailed information about the SLC genes can be found at http://www.bioparadigms.org. Aggregated from references [56,108,109,110]. rBAT, related to b0,+ amino acid transport; NBAT, neutral and basic amino acid transport; 4F2hc, 4F2 cell-surface antigen heavy chain; ATB0,+, Amino acid transporter responsible for the activity of system B0,+; CAT1, cationic amino acid transporter 1; CAT2A/B, cationic amino acid transporter 2A/B; CAT3, cationic amino acid transporter 3; y+LAT1/2, system y+ large amino acid transporter 1/2, b0,+AT, b0,+ amino acid transport; OATP4C1/OATPX/OATP-H, organic anion transport polypeptide 4C1; OCT2, organic cation transporter 2; ORNT1/2/3, ornithine transporter 1/2/3; ORC1/2/3, ornithine transporter 1/2/3; SNAT4/7/8, sodium-coupled neutral amino acid transporters 4/7/8; MATE1, multidrug and toxin extrusion protein 1; P-gp; permeability-glycoprotein; CD98hc, cluster of differentiation 98 heavy chain; FRP, fusion regulatory protein; ATRC1/2/3, human cationic amino acid transporter 1/2/3; ATA3, amino acid transporter A3. E, exchanger (antiporter); F, facilitator (uniporter).

**Table 3:** An overview of tissue distribution of L-arginine and its derivatives transporters on selected organs and tissues (based on mRNA and protein expression).

Organ/Tissue		Transporter
Kidney	Tubular cells (luminal side)	b^0,+^AT-rBAT, MATE1, and P-gp
	Tubular cells (blood side)	CAT1, OCT2, OATP4C1, and y+LAT1-4F2hc
	Unspecified	CAT1
Intestines		ATB^0,+^, OCT2, CAT1, and P-gp
Liver		CAT2A, SNAT4, P-gp, and MATE1
Blood vessel	Endothelial cells	CAT1, CAT2B, y^+^LAT1, and y^+^LAT2
Heart		CAT1
Brain		CAT1, CAT3, y^+^LAT1-4F2hc, ATB^0,+^, OCT2, SNAT7, and SNAT8
Others	Monocytes	y^+^LAT1-4F2hc
	Lymphocytes	CAT2B
	Erythrocytes	y^+^LAT1-4F2hc, y^+^LAT2-4F2hc,
	Placenta	y^+^LAT1-4F2hc, y^+^LAT2-4F2hc, CAT1, CAT2B, CAT3, OCT2, and SNAT4
	Platelets	y^+^LAT1-4F2hc
	Macrophages	CAT2B
	Skin	CAT1
Intracellular	Lysosomes	SNAT7, and SNAT8
	Mitochondria	ORNT1, ORNT2, and ORNT3

For detailed information about the SLC genes, please visit: https://www.ncbi.nlm.nih.gov/gene. Aggregated from references [56,124,125,126].

**Table 4:** Transporters of L-arginine and its derivatives involved in the pathophysiology of the cardiovascular system.

Transporter (Gene/Protein)	Function	Relevance to Pathophysiology of Cardiovascular System
*SLC7A1*/CAT1	Selective supplier of L-arginine to endothelial NOS; involved in interorgan transport of ADMA	NO-mediated endothelial function [12]; reduced activity increases risk for endothelial dysfunction and hypertension [111,112]
*SLC7A2*/CAT2	CAT2A Hepatic uptake of ADMA and SDMA from the circulation. CAT2B uptake of L-arginine by Immune cells.	Downregulation of CAT2 expression by miRNA associated with increased risk for myocardial infarction [142]
*SLC7A-SLC3A2*/y^+^LAT2-4F2hc	High affinity uptake of L-arginine	Loss or a very low activity leads to increase predisposition for CVD [106]
*SLC7A7-SLC3A2*/y^+^LAT1-4F2hc	Mediating cellular ADMA efflux	Decreased expression associated with endothelial dysfunction [143]
*SLC7A9-SLC3A1*/b^0,+^AT-rBAT	Renal tubular reabsorption of L-arginine back into the circulation	Involved in L-arginine homeostasis; loss of function or reduced activity leads to increase risk of hypertension [144,145]
*SLCO4C1*/OATP4C1	Possibly related to renal tubular efflux of ADMA into the urine	Altered expression associated with increased risk of hypertension [146]
*SLC22A2*/OCT2	Mode of action still unknown	Impaired expression associated with essential hypertension [147]

**Table 5:** Characteristics of plasma membrane transporters that accept L-arginine and its derivatives as substrates.

Gene	Protein	Pathophysiological and Clinical Associations	Prototypic Substrate(s)	Prototypic Inhibitor(s)	Species (Expression System)	Evidence for Transport (Direct = D or Indirect = I)			
						L-Arginine	L-Homoarginine	ADMA	SDMA
*SLC6A14;* *SLc6a14*	ATB^0,+^	Susceptibility to secondary diseases in cystic fibrosis [184,185]	Cationic amino acids and neutral amino acids, excluding aspartate and glutamate [43]	Alpha-methyl-DL-tryptophan (α-MT) [50]	Human (human bronchial epithelial cells) [186]	(I) K_M_: 80 ± 8.9 µM	-	-	-
					Human (Xenopus oocytes) [39]	(D) K_M_: 104 ± 35 µM	-	-	-
					Rat (human pneumocytes) [43]	(D) K_M_: 500 ± 110 µM; V_Max_: 33.3 ± 2.1 nmol × mg protein^−1^ × min^−1^	-	-	-
					Rabbit (human cornea) [187]	(D) K_M_: 306 ± 72 µM; V_Max_: 0.12 ± 0.01 nmol × mg protein^−1^ × min^−1^	-	-	-
*SLC7A1; SLc7a1*	CAT1	Knockout mice: lethal [109]; knockdown mice: impaired fetal growth [110]; liver cancer [188]; colorectal cancer [189], breast cancer [190]; hypertension [112,113]	Cationic amino acids (L-arginine, L-ornithine, L-lysine, L-homoarginine, ADMA, SDMA)	N-Ethylmaleimide (NEM) [191]	Human (Xenopus oocytes) [72]	(D) K_M_: 110–160 µM; V_Max_: 1.6–1.8 nmol L-Arg/oocyte/h	-	-	-
					Human (human embryonic kidney/HEK cells) [71]	(D) K_M_: 519 ± 36 µM; V_Max_: 11 ± 0.2 nmol × mg protein^−1^ × min^−1^; IC_50_: 227 µM (inhibitor ADMA)	-	(D) K_M_: 183 ± 21 µM; V_Max_: 26.9 ± 0.8 nmol × mg protein^−1^ × min^−1^; IC_50_: 758 µM (inhibitor L-arginine)	(I) IC_50_: 789 µM (inhibitor L-arginine); 273 µM (inhibitor ADMA)
					Human (HEK cells) [73]	(D) IC_50_: 1320 µM (inhibitor L-homoarginine)	(D) K_M_: 175 ± 7 µM; V_Max_: 12 ± 0.1 nmol × mg protein^−1^ × min^−1^; IC_50_: 1320 µM (inhibitor L-arginine)	(D) IC_50_: 642 µM (inhibitor L-homoarginine)	-
					Human (human fibroblast) [192]	(I) K_M_: 30–200 µM			
					Human (human fibroblast) [193]	-	(I) K_M_: 40 µM	-	-
					Human (human hepatocytes) [57]	-	(I) K_M_: 300 µM	-	-
					Mouse (Xenopus oocytes) [64]	(D) K_M_: 70 µM; V_Max_: 190 nmol L-Arg/oocyte/h	-	-	-
					Mouse (Xenopus oocytes) [194]	(D) K_M_: 77 µM; V_Max_: 0.98 nmol L-Arg/oocyte/h	-	-	-
					Mouse (Xenopus oocytes) [72]	(D) K_M_: 140–250 µM; V_Max_: 1.1–1.6 nmol L-Arg/oocyte/h	-	-	-
					Mouse (human embryonic fibroblast) [154]	(D) K_M_: 107.1 ± 4.1 µM; V_Max_: 0.42 ± 0.01 nmol × mg protein^−1^ × min^−1^	-	-	-
*SLC7A2A*; *SLc7a2A*	CAT2A	L-argininemia [152]	Cationic amino acids (L-arginine, L-ornithine, L-lysine, L-homoarginine, ADMA, SDMA)	NEM [191,195]	Human (Xenopus oocytes) [72]	(D) K_M_: 3360–3,900 µM; V_Max_: 2.2–8.4 nmol L-Arg/oocyte/h	-	-	-
					Human (HEK cells) [123]	(D) K_M_: 3510 ± 419 µM; V_Max_: 19.5 ± 0.7 nmol × mg protein^−1^ × min^−1^	-	(D) K_M_: ~ 3033 ± 675 µM; V_Max_: ~ 11.8 ± 1.2 nmol × mg protein^−1^ × min^−1^	-
					Human (HEK cells) [73]	(D) IC_50_: 3265 µM (inhibitor L-homoarginine)	(D) Saturation not reached	(D) IC_50_: 9244 µM (inhibitor L-homoarginine)	-
					Mouse (xenopus oocytes) [72]	(D) K_M_: 2100–5,200 µM; V_Max_: 3.9–7.1 nmol L-Arg/oocyte/h	-	-	-
*SLC7A2B; SLc7a2B*	CAT2B	L-argininemia [152]	Cationic amino acids (L-arginine, L-ornithine, L-lysine, L-homoarginine, ADMA, SDMA)	NEM [191]	Human (Xenopus oocytes) [72]	(D) K_M_: 320–730 µM; V_Max_: 1.2–4.0 nmol L-Arg/oocyte/h	-	-	-
					Human (Xenopus oocytes) [70]	-			(D) Transported
					Human (HEK cells) [123]	(D) K_M_: 952 ± 92 µM; V_Max_: 15.3 ± 0.4 nmol × mg protein^−1^ × min^−1^	-	(D) K_M_: ~ 4021 ± 532 µM; V_Max_: 14.3 ± 1.0 nmol × mg protein^−1^ × min^−1^	-
					Human (HEK cells) [73]	-	(D) K_M_: 523 ± 35 µM; V_Max_: 11 ± 0.2 nmol × mg protein^−1^ × min^−1^	-	-
					Mouse (Xenopus oocytes) [72]	(D) K_M_: 250–380 µM; V_Max_: 1.1–3.4 nmol L-Arg/oocyte/h	-	-	-
					Mouse (Xenopus oocytes) [196]	(D) K_M_: 187 ± 28 µM	-	-	-
*SLC7A3*; *SLc7a3*	CAT3	Autism spectrum disorder [156]; papillary thyroid carcinoma [197]	Cationic amino acids (L-arginine, L-ornithine, L-lysine)	NEM [191]	Human (Xenopus oocytes) [153]	(D) K_M_: 450 ± 130 µM; V_Max_: 1.4 ± 0.3 nmol L-Arg/oocyte/h	-	-	-
					Mouse (Xenopus oocytes) [155]	(D) K_M_: 40–60 µM; V_Max_: 16 to 60 nmol L-Arg/oocyte/h	-	-	-
					Rat (COS7) [198]	(D) K_M_: 103 ± 12 µM	-	-	-
*SLC7A9*/*SLC3A1*; *SLc7a9*/*SLc3a1*	b^0,+^AT/rBAT	Cystinuria [199]; breast cancer [200] Increased prevalence of hypertension [144,145]	Cystine, Cationic amino acids (L-arginine, L-ornithine, L-lysine), large neutral amino acids (leucine, glutamine)	Heavy metal (lead and mercury) [201]	Human (COS-1 cells) [175]	(I) Transported	-	-	-
					Human (COS-7 cells) [179]	(D) K_M_: 108 µM; V_Max_: 0.65 nmol × mg protein^−1^ × min^−1^	-	-	-
					Human (MDCK cells) [180]	(D) K_M_: 179 µM	-	-	-
					Human (cystinuric patients) [100]	(I) Argininuria	(I) Homoarginiuria	-	-
					Human (Xenopus oocytes) [179]	(D) Influx (K_M_: 85 ± 7 µM; V_Max_: 211 ± 11 nmol L-Arg/oocyte/h); efflux (K_M_: 65 ± 5 µM; V_Max_: 6.1 ± 0.2 nmol L-Arg/oocyte/h)	-	-	-
					Mouse (Xenopus oocytes)[202]	(D) K_M_: 72 ± 35 µM	-	-	-
					Mouse (COS-7) [203]	(D) K_M_: 203 µM; V_Max_: 2570 nmol × mg protein^−1^ × min^−1^	-	-	-
					Rabbit (Xenopus oocytes) [204]	(D) K_M_: 105 µM; V_Max_: 29 nmol L-Arg/oocyte/h	-	-	-
*SLC7A6/* *SLC3A2*	y^+^LAT2-4F2hc	Lysinuric protein intolerance [205]	Neutral amino acids (L-glutamine, L-leucine, L-glycine) and cationic amino acids (L-arginine, L-ornithine, L-lysine)	-	Human (human fibroblast) [205]	(D) K_M_: 145 ± 28 µM; V_Max_: 1479 ± 0.09 μmol × mg protein^−1^ × min^−1^	-	-	-
					Human (Xenopus oocytes) [159]	(D) K_M_: 0.12–0.14 µM	-	-	-
*SLC7A7*/*SLC3A2*; *SLc7a7*/*SLc3a2*	y^+^LAT1-4F2hc	Lysinuric protein intolerance [206]; intrauterine growth retardation [207]; endothelial dysfunction [106]	Neutral amino acids (L-leucine, L-valine) and cationic amino acids (L-arginine, L-lysine, L-ornithine)	-	Human 4F2hc and mouse y^+^LAT1 (oocytes) [162]	(I) Transported	-	-	-
					Human (MDM) [205]	(D) K_M_: 182 ± 35 µM; V_Max_: 3822 ± 0.24 μmol × mg protein^−1^ × min^−1^	-	-	-
*SLC214C1*	OATP4C1	Obesity [208]; endometrial cancer [209]; digoxin disposition in cardiac insufficiency [210]	Amphipathic organic compounds, bromsulphthalein (BSP), bile salts, bilirubin, estrogen conjugates, thyroid hormones, neutral steroid, digoxin, methotrexate	Nicardipine, spironolactone, fluvastatin, crizotinib, levofloxacin, clarithromycin, ritonavir, saquinavir, quinidine, and verapamil [211]	Human (HEK cells) [212]	-	(D) K_M_: 49.9 µM	(D) K_M_: 232.1 µM	-
					Human (madin-darby canine kidney/MDCK cells) [213]	(D) K_M_: 48.1 ± 5.7 μM	(D) K_M_: 49.9 ± 9.6 μM	(D) K_M_: 232.1 ± 78.9 μM	-
*SLC22A2*	OCT2	Potential drug-drug interactions (metformin and cimetidine) [214]	1- methyl-4-phenylpyridinium (MPP+), 4–4-dimethylaminostyryl-N-methylpyridinium (ASP+), histamine, tyramine, metformin [215]	Amitriptyline, doxepin, lansoprazole, pantoprazole, trimipramine [216,217]	Human (HEK cells) [123]	(D) K_M_: > 10,000 µM; V_Max_: > 50 nmol × mg protein^−1^ × min^−1^	-	(D) K_M_: 967 ± 143 µM; V_Max_: 6.3 ± 0.3 nmol × mg protein^−1^ × min^−1^	-
*SLC25A2*	ORC2/ORNT2	Early epileptic encephalopathy [218]; colorectal cancer [219]	Ornithine [220]	Spermine and spermidine [220]	Human (human proteoliposomes) [220] Human (human liposomes) [221]	(D) K_M_: 710 ± 90 µM; V_Max_: 1.2 ± 0.2 μmol × mg protein^−1^× min^−1^	(D) Transported	(D) K_M_: 370 ± 40 µM; V_Max_: 0.31 ± 0.05 nmol × mg protein^−1^ × min^−1^	-
*SLC25A15*	ORC1/ORNT1	Hyperornithinemia hyperammonemia-homocitrullinuria (HHH) syndrome [222]	L-ornithine, L-arginine, carnitine, and citrulline [220]	Citrulline, lysine, spermine, and spermidine [220]	Human (human proteoliposomes) [220]	(D) K_M_: 1580 ± 180 µM; V_Max_: 3 ± 0.4 μmol × mg protein^−1^× min^−1^	(D) Transported	-	-
*SLC25A29*	ORC3/ORNT3	Hyperornithinemia hyperammonemia-homocitrullinuria (HHH) syndrome [223]	L-ornithine, L-arginine, carnitine, and citrulline [224]	Pyridoxal 5’-phosphate, tannic acid, HgCl2, mersalyl, and p-hydroxymercuribenzoate, NEM, and α-cyano-4-hydroxycinnamate [225]	Human (human proteoliposomes) [225]	(D) K_M_: 420 ± 40 µM; V_Max_: 0.237 ± 0.048 μmol × mg protein^−1^× min^−1^	(D) Transported; K_i_: 450 ± 70 µM (inhibitor L-arginine)	-	-
*SLC38A4*	NAT3/ATA3	Placental hypoplasia [226]	Neutral amino acids (L-glutamine, L-leucine, L-glycine) [227]	Alpha-(methylamino) isobutyric acid (MeAIB) [228]	Human (HRPE cells) [229]	(D) K_M_: 300 ± 40 µM; L-arginine inhibited glycine uptake 31%	-	-	-
*SLC38A7*	SNAT7	-	Neutral amino acids (L-glutamine, L-leucine, L-glycine) [227]	(MeAIB) [228]	Human (Xenopus oocytes) [230]	(I) Transported (uptake 4-fold)	-	-	-
*SLC38A8*	SNAT8	Foveal hypoplasia [231]	Neutral amino acids (Lglutamine, L-leucine, L-glycine) [227]	(MeAIB) [228]	Human (Xenopus oocytes) [232]	(I) Transported (uptake 1.4-fold)	-	-	-
*SLC47A1*	MATE1	Potential drug-drug interactions (metformin) [233]	Tetraethylammonium, metformin, salicylic acid, quinine, tenofir, cisplatin [217,233,234]	Cimetidine, imatinib, clonidine, diltiazem, imipramine, ranitidine, chlorhexidine [233,234]	Human (HEK cells) [123]	(D) Transported (uptake ratio 1.3)	-	(D) Transported (uptake ratio 1.1)	
*MDR1/* *ABCB1*	P-gp	Potential drug-drug interactions (digoxin, rifampin, quinidine, St. John’s wort, talinolol, fexofenadine) [235]	Cholesterols, steroid hormones, bilirubin, and numerous drugs (e.g., digoxin, quinidine, ritonavir, etoposide, and dexamethasone) [236,237]	Verapamil, atorvastatin, cyclosporine, progesterone, quinidine, carvedilol, bromocriptine, erythromycin [235,237,238]	Human (MDCK cells) [213]	-	(D) Transported (uptake ratio 1.4 at 50 μM)	(D) Transported (uptake ratio 1.2 at 50 μM)	-

‘–’, not available, not applicable or insufficient information. HEK: human embryonic kidney; MDCK: Madin–Darby canine kidney; HRPE: human retinal pigment epithelial; MDM: monocytes-derived macrophages; KM: Michaelis constant; VMax: maximal velocity of the transport.
